# Hemophagocytic Lymphohistiocytosis: A Rare Complication of COVID-19 in a Patient With Sickle Cell Anemia

**DOI:** 10.7759/cureus.47631

**Published:** 2023-10-25

**Authors:** Rami Al-Handola, Khaled Abdelkader, Arif Karrar, Justine Chinnappan, Geeta Rode

**Affiliations:** 1 Internal Medicine, Hurley Medical Center, Michigan State University College of Human Medicine, Flint, USA; 2 General Medicine, Royal Berkshire Hospital, Reading, GBR; 3 Pulmonology and Critical Care, Hurley Medical Center, Michigan State University College of Human Medicine, Flint, USA

**Keywords:** covid-19, sars-cov-2 associated hlh, secondary hlh, secondary hemophagocytic lymphohistiocytosis (hlh), adult sickle cell anemia, hemophagocytic lymphohistiocytosis (hlh), hlh

## Abstract

Hemophagocytic lymphohistiocytosis (HLH) is an uncommon condition that can be fatal due to overwhelming macrophage activation and cytokine production. It can be primary (familial/genetic) or secondary. It is associated with infections, malignancies, and rheumatologic and immunodeficiency disorders. We report a middle-aged female patient with sickle cell anemia who presented with COVID-19 infection that triggered a vaso-occlusive crisis and resulted in HLH. She had preexisting high ferritin levels and cytopenias, making the diagnosis more challenging. A high index of suspicion and timely treatment is essential to prevent adverse outcomes.

## Introduction

Hemophagocytic lymphohistiocytosis (HLH) is a rare, life-threatening condition resulting from uncontrolled immune system activation, leading to multisystem organ failure [1]. HLH is associated with several infectious etiologies, accounting for almost half of the cases [2], particularly viral infections, accounting for about 70% of those infections [2]. Some of the reported viruses are herpesviruses, adenovirus, influenza, and parainfluenza viruses [2]. It can go undetected as it has features similar to other conditions, such as sepsis and cytokine release syndrome making the diagnosis challenging [3]. In this report, we describe a case of a middle-aged female with sickle cell anemia who presented with a COVID-19 infection that triggered a vaso-occlusive crisis and was subsequently diagnosed with HLH.

## Case presentation

A 54-year-old female with a medical history of sickle cell anemia, iron overload, chronic obstructive pulmonary disease requiring oxygen, paroxysmal atrial fibrillation, hypertension, and chronic kidney disease presented to the emergency department (ED) in Flint, Michigan, in November of 2022 with complaints of intractable bilateral leg pain consistent with a sickle cell crisis. Five days prior to the presentation, the patient had flu-like symptoms and shortness of breath. She was diagnosed with COVID-19 infection and treated with nirmatrelvir/ritonavir.

Physical examination in the ED revealed hypertension with a blood pressure of 178/86 mmHg, tachycardia, and tachypnea. Initial workup revealed anemia, thrombocytopenia, hyperkalemia, acute kidney injury, and elevated liver enzymes. A chest radiograph showed interstitial airspace opacities bilaterally and consolidation in the right lobe of the lung. The patient was admitted and started on broad-spectrum intravenous antibiotics, fluids, and morphine for pain control. The patient deteriorated, became encephalopathic, and hypoxic, eventually requiring intubation and mechanical ventilation. Hematology was consulted, and laboratory data showed anemia, thrombocytopenia, massive elevation of ferritin, and hypertriglyceridemia (Table 1). The patient was also noted to be febrile, which was managed with antipyretics.

**Table 1:** Laboratory data of significance prior to admission, at admission, and discharge

Laboratory test	Prior to presentation	At presentation	At discharge	Normal value
Hemoglobin	7.7 g/dL	5.8 g/dL	6.3 g/dL	12.0-16.0 g/dL
Platelets	181 k/uL	30 k/uL	80 k/uL	130-430 k/uL
Ferritin	3,013 ng/mL	49,381 ng/mL	2,863 ng/mL	10-291 ng/mL
Fibrinogen	441 mg/dL	440 mg/dL	No value	193-473 mg/dL
Triglycerides	79 mg/d	409 mg/d	87 mg/d	​​<150 mg/dL

All these findings were concerning for HLH. Additional laboratory tests were ordered to aid in the diagnosis, including soluble IL-2 receptors and IL-6. Both were elevated at 1239 pg/mL (normal: 175.3-858.2 pg/mL) and 104.5 pg/mL (normal: <6.4 pg/mL), respectively. The diagnosis of HLH was established, and the patient was started on dexamethasone at a dose of 10 mg/day, which increased later to 22 mg/day. The patient was extubated and showed clinical and biochemical improvement (ferritin decreasing and thrombocytopenia resolving). A tapering regimen of steroids was initiated (dexamethasone was reduced to 11 mg/day, to 4 mg/day, to 2 mg/day), and the patient improved but required extensive physical reconditioning and rehabilitation in the community.

## Discussion

HLH is a rapidly progressive, life-threatening, inflammatory disorder characterized by excessive cytokines production, elevated ferritin, and cytopenia [1]. The presentation can be variable, with symptoms of fever, fatigue, rash, lymphadenopathy, hepatosplenomegaly, and multiorgan failure, all of which are thought to be related to the massive cytokine release. HLH can be primarily due to childhood genetic mutations or secondary to an underlying stimulus such as infection, mainly viral infections [2]. The most common triggering viruses are Epstein-Barr virus, parvovirus, human immunodeficiency virus, Hepatitis A, B, and C, influenza, parainfluenza, and adenovirus. Other contributing factors include malignancies, autoimmune, and rheumatological diseases. Prompt diagnosis and treatment are essential for better outcomes and survival [1,3]. However, the diagnosis can be challenging due to the absence of a unique feature [3]. Hemophagocyte observation in bone marrow and the clinical findings associated with HLH can also be observed in other conditions [3].

The diagnosis is based on the HLH 2004 study diagnostic criteria, which include either a molecular diagnosis with mutations of PRF1, STX11, UNC13D, SH2d1A, Munc18-2, Rab27a, or BIRC4. The other available option for diagnosis is by fulfilling five out of the following eight criteria (Table 2) [1,4]. Our patient had at least five of the eight criteria that were not explained by other processes. Those criteria aided in diagnosis and facilitated the initiation of treatment.

**Table 2:** Criteria for diagnosis of HLH in the absence of a genetic mutation based on the HLH 2004 study HLH: Hemophagocytic lymphohistiocytosis

Criteria
The presence of a fever greater than or equal to 38.5°C [1,4]
Presence of splenomegaly [1,4]
Peripheral blood cytopenia ≥2 series: Hemoglobin <9 g/dL, Platelet count <100 ×10^9^/L, Absolute neutrophil count < 1×10^9^/L [1,4]
Hypertriglyceridemia and/or hypofibrinogenemia less than 150 mg/dl [1,4]
Low or absent natural killer cells activity [1,4]
Ferritin greater than 500 ng/mL [1,4]
Hemophagocytosis in bone marrow, spleen, lymph node, or liver [1,4]
Elevated Soluble CD25 (IL2Ra) [1,4]

In the literature, few cases of HLH are reported as a complication of COVID-19 [1-3]. However, there were no reported cases of HLH in a patient with sickle cell anemia who developed COVID-19 infection. HLH and sickle cell disease share some common pathways in their pathophysiology, such as elevated levels of cytokines, interleukin-6, interleukin-2 receptor, and tumor necrosis factor-alpha. HLH treatment may require immunosuppressive therapy, and in some cases, hematopoietic stem cell transplantation. If left untreated, HLH is usually fatal [5]. However, the rare phenomenon of spontaneous resolution of this illness is also reported. The response of our patient to immune suppressive therapy also aided in confirming the diagnosis. High suspicion, early recognition, and differentiating HLH from similar conditions lead to favorable outcomes [6]. To the best of our knowledge, this case is the first reported presentation of HLH in a sickle cell patient who developed a COVID-19 infection.

## Conclusions

The objective of this case report is to consider the diagnosis of HLH, which can mimic other chronic conditions such as sickle cell anemia and can be fatal if left untreated. Similarities should be carefully considered when dealing with complex, critically ill patients. Using scoring systems can aid in the diagnosis, leading to the initiation of the appropriate treatment.
